# High-Performance All-Organic DFB and DBR Waveguide Laser with Various Grating Height Fabricated by a Two-Photon Absorption DLW Method

**DOI:** 10.1038/s41598-019-47098-4

**Published:** 2019-07-22

**Authors:** Naoto Tsutsumi, Keiichi Kaida, Kenji Kinashi, Wataru Sakai

**Affiliations:** 1 Faculty of Materials Science and Engineering, Kyoto Institute of Technology, Matsugasaki, Sakyo, Kyoto 606-8585 Japan; 2 Master’s Program of Innovative Materials, Graduate School of Science and Technology, Kyoto Institute of Technology, Matsugasaki, Sakyo, Kyoto 606-8585 Japan

**Keywords:** Laser material processing, Surfaces, interfaces and thin films, Micro-optics

## Abstract

Organic solid-state lasers (OSSLs) with distributed feedback (DFB) structures or distributed Bragg reflectors (DBRs) are promising for potential application in bio-sensing and hazardous materials detection. Here, the laser performances of the all-organic DFB waveguide lasers with various grating heights ranging from 0.4 to 4.7 μm were investigated. The grating structures used as the lasing cavity were fabricated using a two-photon absorption (TPA) direct laser writing (DLW) method with an SU-8 negative photoresist. The laser active layer consisted of a rhodamine 6G (R6G) laser dye and a cellulose acetate (CA) matrix. The R6G/CA solution was spin-coated onto the quartz substrate with the cavity (grating) structures to fabricate the DFB waveguide laser devices. The diffraction order of lasing ranged from *m* = 4 to 7. As the grating height was increased to 1.9 μm, the slope efficiency increased for all diffraction orders and the threshold decreases for each diffraction order. The dependence of the cavity (grating) length on the laser performances was investigated. The slope efficiency increased as the cavity length increased to 300 μm. The effect of the cavity (grating) position on the slope efficiency and the threshold position of the cavity (grating) was also studied. A maximum slope efficiency of 10.2% was achieved for the DFB waveguide laser device with a cavity (grating) length of 300 μm, a cavity position at 6 mm from the emission edge of the waveguide, and an aspect ratio ≈3 between the grating height of 1.74 μm and the grating width of 0.6 μm for the diffraction order *m* = 6 for lasing.

## Introduction

Organic solid-state lasers (OSSLs) have been extensively investigated in the past two decades^1–3^. The OSSL distributed feedback (DFB) laser can be applied to bio-sensors^4^ and sensors for solvent elimination^5^. Organic laser dye-doped polymers and conjugated fluorescent polymers are commonly used to fabricate OSSL devices. The advantage of organic materials is the easy fabrication of the laser active waveguide using drop casting or spin-coating. Furthermore, the organic photoresist materials can be easily used for the fabrication of the sequence of the microcavity for the DFB and the distributed Bragg reflector (DBR) structures. Interference of two laser beams can be used to fabricate the sequence of the grating structures for DFB laser devices^6–11^. The slope efficiency and the threshold for lasing are the key parameters for the light-pumped DFB lasers. A high slope efficiency of 12% was reported for the organic DFB waveguide laser device with an active layer dispersed by SiO_2_ nanoparticles with rhodamine 6G (R6G) laser dye^12^. Greyscale electron beam lithography was applied to fabricate first diffraction order DFB laser device with R6G doped SU-8 negative photoresist^13^. The roll-to-roll method for the fabrication of multilayered sheet organic DFB laser was also reporte^14^.

In our previous report^8^, the dependence of the grating height less than 80 nm on the threshold and the slope efficiency for lasing were investigated for the various orders of the diffraction process (*m*) ranging from *m* = 1 to 3; it was found that the slope efficiency increases with increasing grating height, and lower threshold was measured for lower diffraction orders. The highest slope efficiency of 2% was achieved for the device with the grating height of 30 nm for the first-order gratings, *m* = 1. The interference of two light beams was used to fabricate the grating structures. Unfortunately, the interference method limits the grating height. The grating height for *m* = 1 ranged from 5 to 30 nm, while that for *m* = 2 ranged from 8 to 30 nm, and that for *m* = 3 ranged from 15 to 60 nm^8^.

Direct laser writing (DLW) through two-photon excitation has been widely used for the fabrication of micro-scale structures such as micro-lens arrays^15^, three-dimensional microstructures^16^, gold microstructures^17^, and metamaterials^18^. DLW through two-photon polymerization can fabricate a deeper grating height using a negative photoresist. Two-photon polymerization of R6G-doped SU-8 negative photoresists by a DLW method obtained a DFB organic laser device with the grating height of 80 nm and the grating pitch of 550 nm, corresponding to the third diffraction order (*m* = 3)^19^. A slope efficiency <1% was obtained^19^. Here, we need to consider the diffraction limit for the DLW method. The linewidth of the structures fabricated by two-photon polymerization is limited by the diffraction limit through two-photon absorption. Thus, the linewidth of the fabrication limits the grating pitch and grating width of the DFB structures and, therefore, the order of the diffraction process for lasing. In the present case, as shown in Fig. S1 in the supplementary information, the minimum linewidth is 460 nm, so *m* = 4 is the minimum order for the diffraction process using the present DLW method. In the present study, the dependence of the cavity length of the grating, the grating height, and the grating pitch related to the order on the DFB laser performances were investigated. Grating structures corresponding to a diffraction order varying from *m* = 4 to 7 were fabricated using a SU-8 negative photoresist. The grating height ranged from 0.4 μm to 4.7 μm, and the cavity (grating) length ranged from 48 μm to 1600 μm. Grating pitch, one cycle of the grating structure, ranged from 800 nm to 1400 nm, corresponding to a diffraction order variation from *m* = 4 to 7. The effects of grating height, cavity (grating) length, and grating position on the laser performances of the threshold and the slope efficiency were investigated for the DFB laser waveguide devices consisting of the grating structures of an SU-8 negative photoresist and a laser active layer of the R6G-doped cellulose acetate (CA).

## Experimental Section

### Materials

SU-8 50 and SU-8 2002 negative photoresists (Microchem, USA) were used for fabricating the grating structures as a laser cavity. SU-8 consisted of an epoxy monomer, photo acid, and solvent. R6G (Aldrich Chem., USA) was used as the laser dye, and CA (Aldrich Chem., USA) was used as the waveguide matrix to dissolve the R6G laser dye. Diacetone alcohol (DAA (Nacalai Tesque, Japan) was used as the solvent for preparing the CA waveguide with the R6G laser dye.

### Fabrication of grating structures for DFB

Either SU-8 50 or SU-8 2002 was spin-coated onto a quartz substrate. Prior to laser illumination, a spin-coated sample film was pre-baked at 65 °C for 15 min, followed by soft-baking at 95 °C for 20 min to evaporate the solvent.

**Figure 1:**
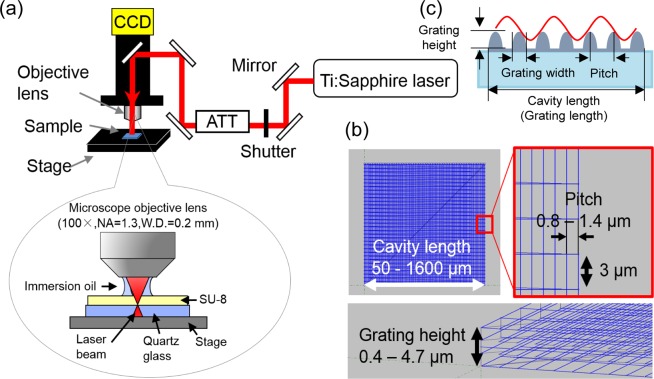
(**a**) Schematic diagram of the apparatus for the TPA DLW method. (**b**) 3D drawing images on a computer. (**c**) Obtained grating structures.

The DLW method through a two-photon absorption (TPA) was used to fabricate the grating structures using an SU-8 negative photoresist under a microscope. A schematic diagram of the TPA DLW apparatus is shown in Fig. 1(a). A Ti:Sapphire femtosecond pulse laser (MaiTai, Spectra-Physics, USA) delivering 100 fs pulse width at 800 nm with a repetition rate of 80 MHz was used as the laser source. The laser beam emitted from MaiTai was introduced into a microscope apparatus (Olympus BX611WI) through an attenuator (ATT) and focused onto the sample film with an oil-immersion objective lens (Olympus UPlanFLN lens, 100x, NA = 1.30). The laser intensity was attenuated by an ATT. The three-dimensional grating structures were fabricated in an SU-8 negative photoresist using a three-dimensional stage (Newport VP-25XA-XYZ, USA) controlled by an ALPS 3861. The resolution of the stage movement was 100 nm. Three-dimensional drawing images produced by a computer are shown in Fig. 1(b). The grating height, width, and pitch, and the cavity (grating) length are defined in Fig. 1(c).

After laser illumination, the SU-8 negative photoresist was baked at 65 °C for 1 min followed by baking at 95 °C for 4 min to complete the cross-linking reaction. Regions not cross-linked were removed with an SU-8 developer (Microchem, USA) for 7 min. After development, the fabricated sample was rinsed with 2-propanol (Nacali Tesque, Japan), followed by drying at ambient conditions, and was hard-baked at 175 °C for 60 min.

### Microscope measurements

The fabricated grating structures were measured using a scanning electron microscope (SEM, Hitachi S-3000, Japan), an atomic force microscope with a close contact (AFM, Pacific Nanotechnology Nano-R, USA), and a digital microscope (Hirox KH-8700, Japan). Prior to the SEM measurement, conductive platinum was sputtered for 120 s on the surface of the object using an ion-sputtering apparatus (Hitachi E-1010, Japan). The thickness of the sample film was measured using an AFM apparatus.

**Figure 2:**
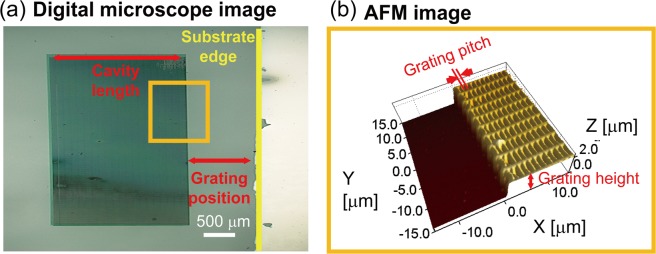
(**a**) CMOS image of the fabricated gratings observed by a digital microscope. (**b**) AFM image of the fabricated gratings.

As shown in the supporting information, support walls (interval of either 3 μm or 1.2 μm) were initially built, and then the line structures for the gratings were fabricated laterally. The digital microscope image of the fabricated cavity for the DFB structures and the AFM image of the fabricated gratings inside the cavity are shown in Fig. 2.

### Fabrication of the laser active waveguide

A 10 wt% DAA solution of CA with 0.5 wt% R6G laser dye (R6G/CA 0.5/99.5 by wt.) was spin-coated on a quartz substrate with a cavity (grating structures). The spin-coated waveguide was dried at 80 °C for 60 min to remove the solvent.

### Characterization

Absorption spectra of the spin-coated film and the SU-8 solution in a quartz cell were recorded using a Shimadzu UV-2100 UV-vis spectrometer. The fluorescence spectrum of the spin-coated film was measured using a Shimadzu RF-1500 fluorophotometer.

### Laser emission measurements

Laser emission spectra emitted from the edge of the waveguide device pumped by a laser source were monitored using a Tokyo Instruments multichannel spectrometer equipped with 1200 line/500 nm gratings and an Andor iDus charge-coupled device. The resolution of the multichannel spectrometer was 0.2 nm. An Nd:YAG laser delivering 30 ps pulse duration at 532 nm with a 10 Hz repetition rate was used as the pump laser source (Ekspla PL2403, Lithuania). A cylindrical lens (*f* = 300 nm) was used to change the circular laser beam with a diameter of 6.5 mm to a stipe-shaped excitation beam with a length of 6.5 mm and width of 33 μm. To monitor the excitation beam intensity using an Ophir photodiode-type pyrometer PD10 with a Nova II Display, the excitation beam was reflected by a half mirror before the waveguide sample film. An optical-fibre coupled photodiode pyrometer (Ophir PD-10-P) with a Nova II Display equipped with an optical filter to cut out the incident excitation beam was used to monitor the absolute intensity of the laser emission from the edge of the waveguide.

## Results and Discussion

### 100 μm × 100 μm square cavity for *m* = 4, 5, 6, and 7

**Figure 3:**
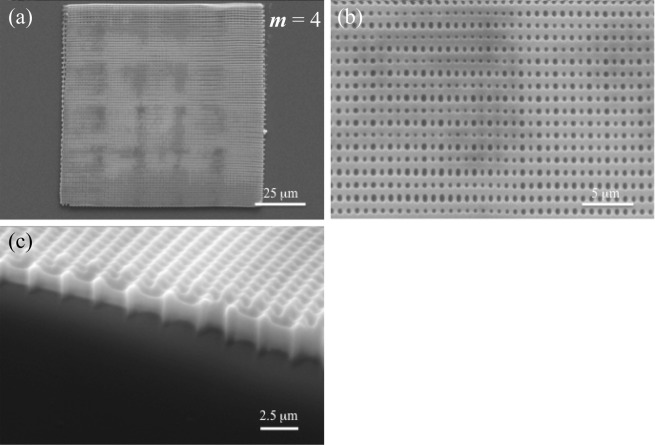
SEM images for the gratings corresponding to *m* = 4. (**a**) Entire SEM image × 800, (**b**) enlarged SEM image × 4000, and (**c**) SEM image of the edged part with 60 degree tilting × 6000.

**Figure 4:**
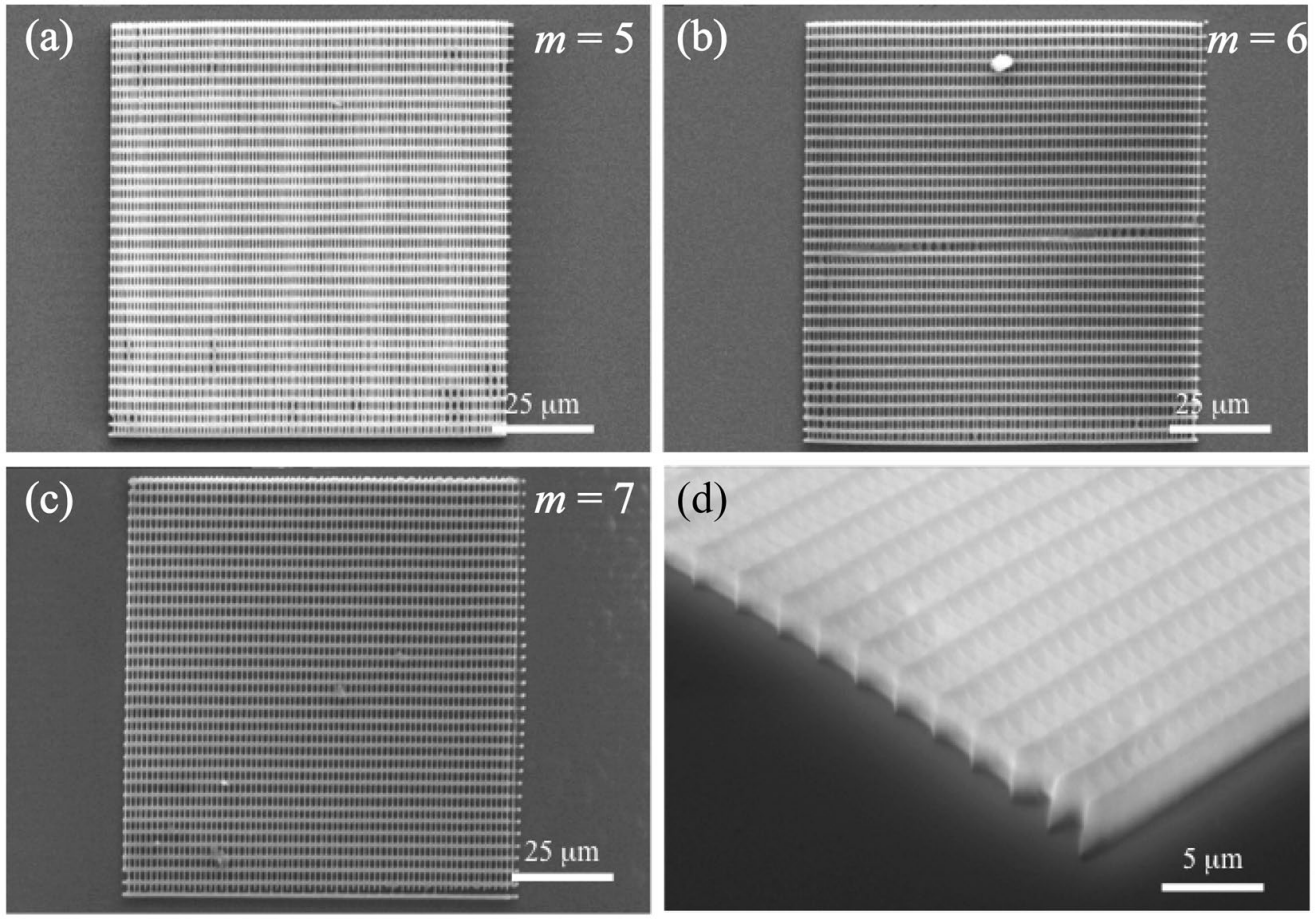
SEM images for the gratings corresponding to *m* = 5–7. (**a**) Entire SEM image for *m* = 5, (**b**) that for *m* = 6, (**c**) that for *m* = 7 × 800, and (**d**) SEM image of the edged part with 60 degree tilting × 6000.

To build the long ranged standing gratings, the support walls must be fabricated. Based on the results in Fig. S2 in the supplementary information, the distance of the support walls is 1.2 μm for *m* = 4, and is 3 μm for *m* = 5, 6 and 7. DLW conditions are summarized in Table 1. Figures 3 and 4 show SEM images of 100 μm × 100 μm square cavities for *m* = 4 and *m* = 5–7, respectively. The SEM images clearly show that the proper grating structures (grating length of 100 μm) for the laser cavity are formed using a SU-8 negative photoresist.

**Table 1:** DLW conditions.

*m*	Laser Intensity (mW)	Scanning Rate (μm s^−1^)
**(a) DLW conditions of the grating lines**		
4–7	7	40
**(a) DLW conditions of the support walls**		
4	9	40
5–7	14.7	50

Therefore, the cavity (grating) length of the DFB structures was fixed to 100 μm and the cavity (grating) position was set at 4 mm from the emission edge of the waveguide. After spin-coating the laser active (R6G/CA) solution onto the cavity, the DFB laser device was fabricated. The emission wavelength of the DFB laser device (lasing wavelength, *λ*_L_) is related to the grating pitch and the effective refractive index in the waveguide:

1 $${\lambda }_{L}=\frac{2{n}_{{\rm{eff}}}{\Lambda }_{{\rm{th}}}}{m},$$

where *n*_eff_ is the effective refractive index of the waveguide, *Λ*_th_ is the grating pitch in the cavity, and *m* is the diffraction order (order number, integer) for lasing depending on the length of the grating pitch.

### Waveguide parameters

Waveguide parameters of the guided mode, the effective refractive index, and the optical confinement in a four-layer waveguide (Air/Laser active layer of R6G doped CA/SU-8 grating structure/quartz substrate) were calculated using NL Guide Ver. 5.16 waveguide simulator. The typical plots of the optical confinement and the effective index as a function of thickness are summarized for various thicknesses of SU-8 for the grating structures in Fig. S3 in the supplementary information.

### Grating height dependence

The feedback gain for DFB lasing (*G*) is the product of the coupling coefficient (*κ*) and the cavity length (resonance length, *L*):

2 $$G=\kappa \cdot L$$

The coupling of the waves is defined by the coupling coefficient (*κ*):

3 $$\kappa =\frac{\pi h({n}_{{\rm{f}}}^{2}-{n}_{{\rm{eff}}}^{2})}{{\lambda }_{{\rm{L}}}{h}_{{\rm{eff}}}{n}_{{\rm{eff}}}}$$

4 $${h}_{{\rm{eff}}}\approx \frac{\lambda }{\sqrt{{n}_{{\rm{f}}}^{2}-{n}_{{\rm{s}}}^{2}}}$$

where *h* is the grating height, *n*_f_ is the refractive index of the active layer (waveguide), *n*_s_ is the refractive index of the substrate, and *h*_eff_ is the minimum effective film thickness of the fundamental mode.

According to Eqs (2) and (3), the feedback gain for DFB lasing (*G*) is proportional to the grating height (*h*) and the cavity length (*L*):

5 $$G\propto h\cdot L$$

Thus, the grating height and cavity length are important parameters for the DFB laser.

In our previous studies^6–11^, DFB structures were fabricated using a negative photoresist and the laser performances of the threshold and the slope efficiency were investigated. For *m* = 1, the grating pitch is 200 nm and the grating width is 100 nm, and as *m* is increased, the grating pitch is 200 × *m* and the grating width is 100 × *m*. For *m* = 1, 2, and 3, holographic interference beams were used to fabricate the gratings with the widths ranging from 100 to 300 nm.

**Figure 5:**
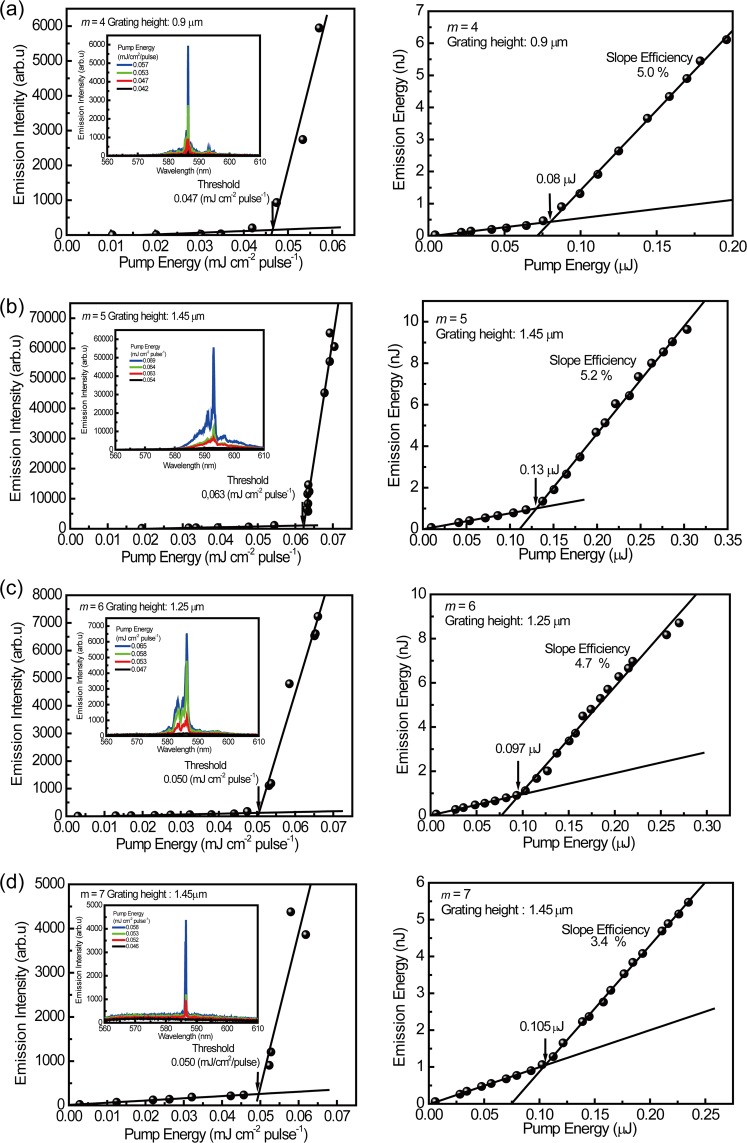
Lasing threshold, laser emission spectrum, and slope efficiency for lasing. Left figure: plots of emission intensity as a function of pump energy. Intersection gives the threshold for lasing. Right figure: plots of emission energy as a function of pump energy. The slope gives the slope efficiency for lasing. Inset figure in the left side shows the laser emission spectra. (**a**) *m* = 4, grating height = 0.9 μm, (**b**) *m* = 5, grating height = 1.45 μm, (**c**) *m* = 6, grating height = 1.25 μm, and (**d**) *m* = 7, grating height = 1.45 μm. The cavity length of the DFB grating structure is fixed at 100 μm, and the cavity (grating) position is fixed at 4 mm from the emission edge the waveguide.

Table 2 summarizes the thickness of the laser active layer (R6G/CA) on the DFB structures, the guided mode, effective index (*n*_eff_), optical confinement, lasing wavelength, threshold, and the slope efficiency for lasing for the waveguide laser device with various grating height and pitch values corresponding to the diffraction modes *m* = 4, 5, 6, and 7. The grating pitch, height and thickness, and the lasing wavelength, threshold, and slope efficiency were measured for each waveguide DFB laser device. The effective refractive index and the optical confinement were calculated using NL Guide software for the lasing wavelength. As the grating height increased, the emitted light was confined in the higher guided mode with the appropriate effective refractive index. Figure 5 shows examples of the plots of the emission intensity and the emission energy as a function of pump energy for the laser devices with various grating pitches corresponding to *m* = 4, 5, 6, and 7. The lasing spectrum is shown in the inset in the left figure; peak at 586. 5 nm for *m* = 4, that at 593.8 nm for *m* = 5, that at 586.4 nm for *m* = 6, and that at 586.4 nm for *m* = 7 are measured. In addition to these main peaks, some additional small peak is appeared. Left side peak at ca. 591.1 nm for *m* = 4 in Fig. 5(b) is due to the lasing emission at TE_4_ guided mode (*n*_eff = _1.4710). Amplified spontaneous emission (ASE) is also overlapped in Fig. 5(c). For higher order modes (TE_8_ and TE_12_), lasing performances are also checked. Single laser emission for TE_8_ guided mode was measured with no mode competition. Laser emission at 582.5 nm for TE_12_ guided mode (*n*_eff_ = 1.4693) is competed with other emission peak due to TE_11_ guided mode (*n*_eff_ = 1.4768), and that due to TE_10_ guided mode (*n*_eff_ = 1.4826).

**Table 2:** Summary of the thickness of laser active layer (R6G/CA) on DFB structures, guided mode, effective refractive index (*n*_eff_), optical confinement, lasing wavelength, threshold, and slope efficiency for lasing for waveguide laser devices with various grating pitch and height values.

Pitch^a^ (nm)	Pitch^b^ (nm)	Grating height (μm)	Thick-ness (μm)	Guided mode	*n*_eff_	Optical confine-ment	Lasing wavelength (nm)	Threshold (mJ cm^−2^ pulse^−1^)	Slope efficiency (%)
***m*** = **4**									
812	792.1	0.6	1.5	TE_2_	1.4716	0.845	582.8	0.061	3.2
784	794.8	0.8	1.45	TE_2_	1.4745	0.933	586.0	0.045	3.9
797	794.2	0.9	1.5	TE_2_	1.4769	0.889	586.5	0.047	5.0
***m*** = **5**									
1013	994.3	0.55	1.5	TE_2_	1.4688	0.717	584.3	0.071	2.6
1001	995.2	0.8	1.5	TE_2_	1.4751	0.939	587.2	0.047	4.0
1004	1002.6	1.45	1.9	TE_3_	1.4806	0.836	593.8	0.063	5.2
***m*** = **6**									
1207	1198.4	0.5	1.5	TE_2_	1.4642	0.586	584.9	0.059	1.9
1202	1192.1	0.8	1.5	TE_2_	1.4752	0.939	586.2	0.059	2.8
1198	1182.4	0.9	1.5	TE_2_	1.4772	0.883	582.2	0.068	3.3
1204	1189.5	1.1	1.56	TE_3_	1.4736	0.867	584.3	0.038	2.9
1192	1194.9	1.25	1.3	TE_3_	1.4720	0.882	586.4	0.050	4.7
1204	1202.2	2.8	1.4	TE_6_	1.4760	0.695	591.5	0.087	5.2
1177	1195.6	3.5	1.4	TE_8_	1.4726	0.791	586.9	0.068	2.5
1231	1189.3	4.7	2.65	TE_12_	1.4693	0.737	582.5	0.115	2.0
***m*** = **7**									
1380	1390.7	0.4	1.4	TE_1_	1.4751	0.935	586.1	0.1	1.1
1381	1394.1	1.45	1.88	TE_4_	1.4721	0.528	586.4	0.050	3.4
1412	1400.7	1.7	1.5	TE_4_	1.4745	0.897	590.1	0.060	3.5

^a^Average pitch measured using an AFM.

^b^Calculated using Eq. (1).

The threshold is the minimum input energy for lasing that is given by the intersection of the two lines in the left figure. Slope efficiency is shown in the pump energy region above the threshold in the right figure. Using the same approach as shown in Fig. 5, the threshold and the slope efficiency were measured for the DFB laser devices with various grating heights for *m* = 4, 5, 6, and 7.

**Figure 6:**
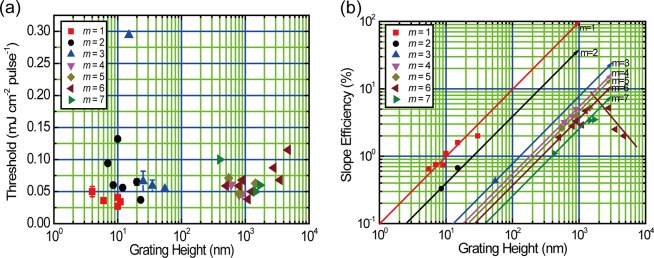
Plots of (**a**) threshold and (**b**) slope efficiency as a function of the grating height. Thresholds and slope efficiencies for *m* = 1, 2, and 3 were taken from the literature^8^.

The resultant threshold and slope efficiency for lasing are plotted as a function of the grating height in Fig. 6(a,b), respectively. The 82% amount of the threshold ranged from 0.038 to 0.071 mJ cm^−2^ pulse^−1^ with an average of 0.056 mJ cm^−2^ pulse^−1^ irrespective of the grating height, as shown in Table 2 and Fig. 6(a). Comparing the thresholds at the same diffraction order from 1 to 7, the threshold decreases with increasing the grating height except for the case for the grating height above 2 μm. These results are consistent with Eq. (5).

On the other hand, the slope efficiency depends strongly on the grating height, as shown in Fig. 6(b). The data for *m* = 1, 2, and 3 are taken from our previous report^8^. For *m* = 1, 2, 3, 4, 5, 6, and 7, the slope efficiency increases with increasing grating height except for the decrease in the slope efficiency at the grating height greater than 1.9 μm (1900 nm) for *m* = 6.

The results presented in Fig. 6(b) imply that an increase in the grating height for each diffraction order leads to the increased feedback gain for DFB lasing. Namely, the grating height is linearly proportional to the coupling coefficient, which is consistent with the relation in Eq. (3). However, it is difficult to compare the slope efficiency for the different values of *m*. Therefore, to unify the different diffraction orders, the aspect ratio between the grating height and grating width was adopted.

**Figure 7:**
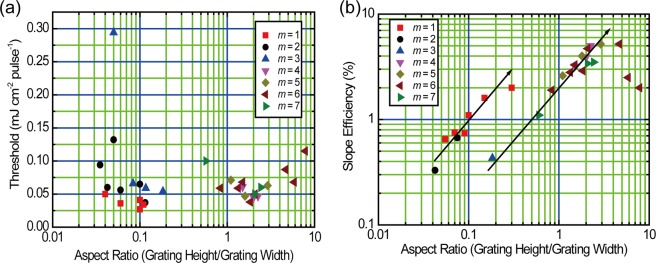
Plots of (**a**) threshold and (**b**) slope efficiency as a function of the aspect ratio between grating height and grating width.

The threshold and the slope efficiency are replotted as a function of the aspect ratio between the grating height and the grating width as shown in Fig. 7. The slope efficiencies for *m* = 1 and 2 are categorized in one group, and those for *m* = 3, 4, 5, 6, and 7 in another group. For both cases, the slope efficiency linearly increases with increasing aspect ratio. The slope efficiency for the diffracion orders higher than *m* = 3 show a unified trend based on the aspect ratio between the grating height and grating width irrespective of the different diffraction order, and the aspect ratio of 3 gives the maximum slope efficiency. At aspect ratios larger than 3, the slope efficiency is decreased.

At diffraction orders higher than *m* = 2, the emission loss of the feedback light from the waveguide (radiation loss) should be considered. Indeed, as shown in Fig. 6(b), the slope efficiency is decreased as the diffraction order increases at the same grating height.

### Cavity (grating) length dependence

**Figure 8:**
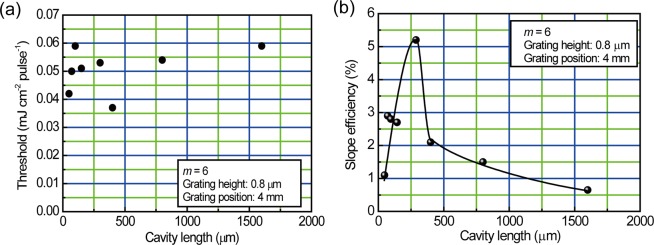
Plots of (**a**) threshold and (**b**) slope efficiency as a function of the cavity length. Solid curve in the figure is a guide for the eye.

Eqs (2) and (5) predict that the feedback gain for DFB lasing is proportional to the cavity length (*L*) equivalent to the grating length. We fabricated different samples for which the cavity length ranged from 48 μm to 1600 μm. The plots of the threshold and the slope efficiency as a function of cavity length are shown in Fig. 8. The slope efficiency increases with increasing cavity length up to 300 μm because greater cavity length leads to larger feedback gain.

### Cavity (grating) position dependence

**Figure 9:**
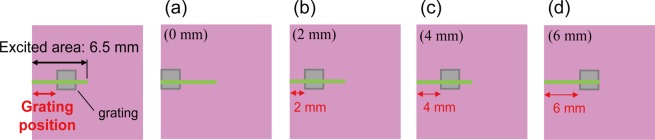
Schematic illustration of the grating position in the waveguide. The excited (pumped) area is 6.5 mm, equivalent to the diameter of the pump laser. The cavity (grating) length is 400 μm, the grating height is 0.8 μm, the grating pitch is 1200 nm, and the grating width is 600 nm.

**Figure 10:**
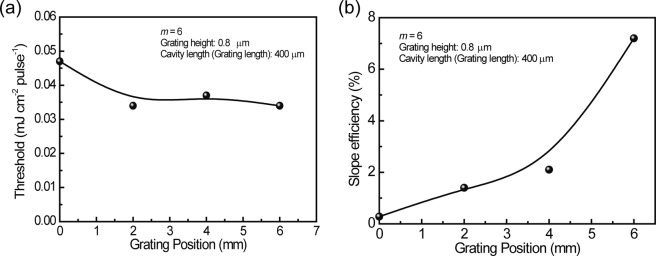
Plots of (**a**) threshold, (**b**) slope efficiency as a function of the grating position. Solid curves in the figure are provided as a visual guide.

In the above discussion, the cavity (grating) position is fixed at 4 mm from the emission edge of the waveguide. Here, the dependence of the cavity (grating) position on the threshold and the slope efficiency was investigated. A schematic illustration of the cavity (grating) position in the waveguide is presented in Fig. 9. The cavity (grating) length is 400 μm, the grating height is 0.8 μm, and *m* = 6 (grating width of 600 nm and grating pitch of 1200 nm). The cavity (grating) position is changed from 0 to 6 mm by 2 mm increments, obtaining (a) 0 mm, (b) 2 mm, (c) 4 mm, and (d) 6 mm. The excited area is exposed to the rectangular shape laser beam (6.5 mm length × 0.033 mm width). Thus, the lasing active area of the exposed area is varied by the position of the cavity (grating). The threshold and the slope efficiency are plotted as a function of the cavity (grating) position in Fig. 10. As shown in Fig. 10(a), the threshold at 0 mm cavity (grating) position is 0.047 mJ cm^−2^ pulse^−1^ and it plateaus at 0.034 mJ cm^−2^ pulse^−1^ above the 2 mm cavity (grating) position. On the other hand, as shown in Fig. 10(b), the slope efficiency increases with the increase in the cavity (grating) position from the emission edge. This means the increase of feedback gain in the active layer with the increase in the cavity (grating) position from the emission edge. The slope efficiency of 7.2% was measured at the cavity (grating) position of 0.6 cm (6 mm).

### Maximum slope efficiency

**Figure 11:**
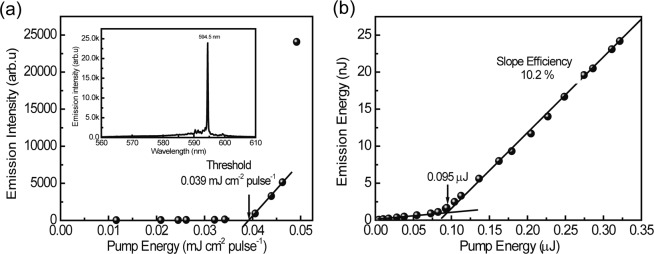
(**a**) Pump energy dependence of the peak intensity at 594.5 nm and (**b**) emission energy as a function of pump energy. The slope of the plot of emission energy versus the pump energy gives the slope efficiency.

As discussed above, the appropriate grating parameters were evaluated as follows: the cavity (grating) position is at 6 mm from the emission edge of the waveguide laser device, the cavity (grating) length is 300 μm, and the aspect ratio between grating height 1.74 μm and grating width 0.6 μm is 3 for the diffraction mode of *m* = 6. The resultant threshold, laser emission spectrum, and slope efficiency are shown in Fig. 11. The threshold of 0.039 mJ cm^−2^ pulse^−1^ and the slope efficiency of 10.2% were measured for the R6G/CA waveguide laser device. The slope efficiency of 10.2% is higher than the previously reported values of 2.0%^8^, 3.4%^6^, and 8.0%^14^ and close to 12%^12^ for the same R6G/CA waveguide type DFB laser devices. In this DFB device geometries, the corrugated area (grating underneath) acts as a waveguide DBR mirror for the gain medium of active layer in the uncorrugated area (no grating underneath) located between the waveguide edge and the waveguide DBR mirror. In such geometries, the optical loss due to radiation mode from the active layer in the uncorrugated area is reduced^2^. This is one reason for the high slope efficiency of 10.2%.

## Conclusions

The dependence of the grating height, cavity (grating) length, and the position of the cavity on the threshold and the slope efficiency for lasing were investigated for the DFB waveguide laser devices consisting of an SU-8 negative photoresist as the grating structures and the R6G doped CA matrix as the laser active layer. The grating height ranged from 0.4 to 4.7 μm. The cavity (grating) length varied from 48 to 1600 μm. The cavity (grating) position varied from 0 mm to 6 mm from the emission edge of the laser waveguide. Diffraction order for lasing varied from *m* = 4 to 7. These grating parameters affect the threshold for lasing and have a strong effect on the slope efficiency of lasing. The threshold ranged from 0.038 to 0.071 mJ cm^−2^ pulse^−1^, and the average threshold was 0.056 mJ cm^−2^ pulse^−1^. An increase in the grating height to 1.9 μm increases the slope efficiency. An increase in the cavity (grating) length to 300 μm increases the slope efficiency. The position of the cavity (grating) from the emission edge of the waveguide is strongly related to the lasing performance characteristics. The maximum slope efficiency of 10.2% was measured for the DFB waveguide laser device with a grating height of 1.74 μm, an aspect ratio between the grating height and width of 3, a cavity (grating) length of 300 μm, and the position of the cavity (grating) at 6 mm from the emission edge of the waveguide.

## Supplementary information

Supplementary Information
