# Supplementary material for: High-Performance All-Organic DFB and DBR Waveguide Laser with Various Grating Height Fabricated by a Two-Photon Absorption DLW Method
Source: Sci Rep. 2019 Jul 22;9:10582. doi: 10.1038/s41598-019-47098-4 (PMC6646654; doi:10.1038/s41598-019-47098-4)
Supplement: Supplementary file 1 — Supplementary Information [file 41598_2019_47098_MOESM1_ESM.doc]

Supplementary Information

**High-Performance All-Organic DFB and DBR Waveguide Laser with Various Grating Height Fabricated by a Two-Photon Absorption DLW Method**

Naoto Tsutsumi,^1^* Keiichi Kaida,^2^ Kenji Kinashi,^1^ Wataru Sakai,^1^

^1^ Faculty of Materials Science and Engineering

^2^ Master’s Program of Innovative Materials, Graduate School of Science and Technology

Kyoto Institute of Technology, Matsugasaki, Sakyo, Kyoto 606-8585, Japan

**Post Baked Conditions for Narrowed Line Width**

Post baked condition is explored to achieve the minimum line width. **Table S1** summarizes the post baked condition for narrowing the line width. Line width is plotted as a function of laser intensity for each condition in **Figure S1**. The minimum line width of 0.46 μm was achieved for the condition B, laser intensity of 6 mW, post baked at 65 °C for 1 min followed by at 95 °C for 4min.

**Table S1.** Summary of post baked time for narrowing the line width.

| Condition | 65 ℃ (min) | 95 ℃ (min) |
| --- | --- | --- |
| A | 10 | 10 |
| B | 1 | 4 |
| C | 1 | 2 |


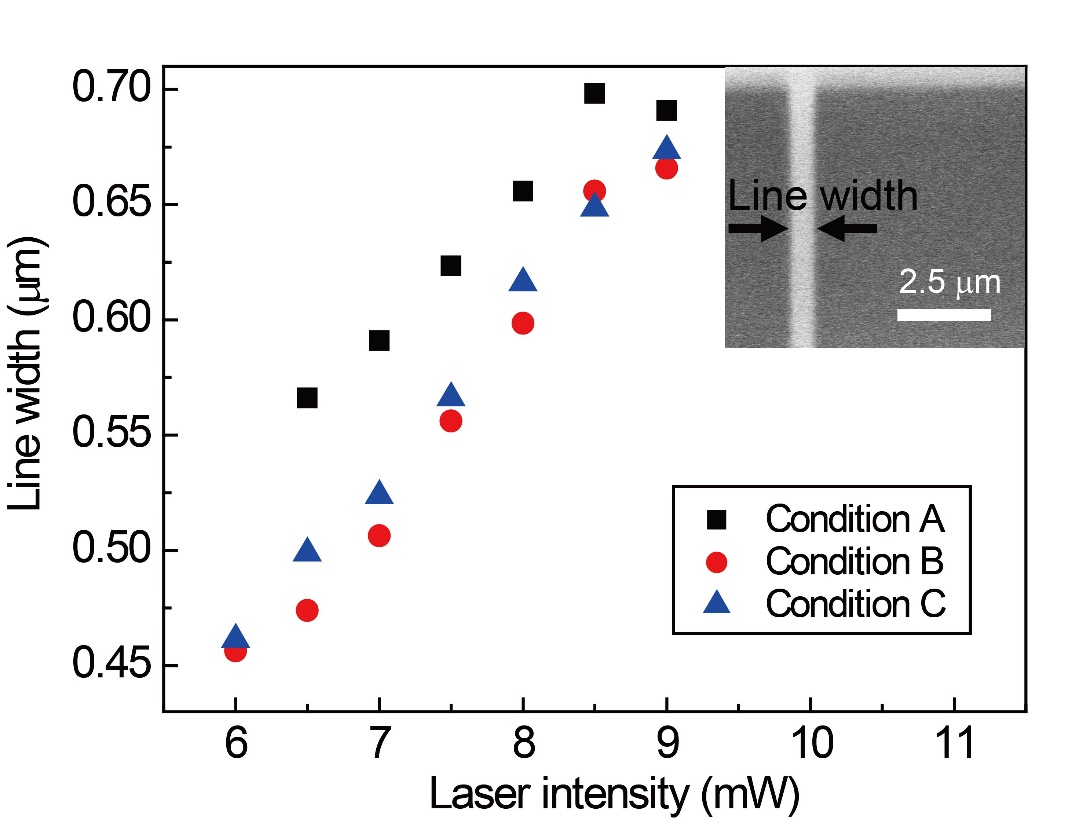


**Figure S1.** The plots of line width as a function of laser intensity for each condition**.**

**Support Walls and Grating Structures**

The relation between the distance between supporting walls and grating structures corresponding the diffraction order ranging from *m* = 3 to 9 was investigated. Without support walls, grating structures were difficult to fabricate. Support walls with length of 300 μm with intervals of 2, 3, and 5 μm are first fabricated. Then grating structures with intervals of 600, 800, 1000, 1200, 1400, 1600, and 1800 nm corresponding to *m* = 3, 4, 5, 6, 7, 8, and 9 are fabricated. Drawing illustration is shown in **Figure S2(a)** and the SEM image of the obtained structures is shown in **Figure S2(b)**. For m = 3 (line distance = 600 nm), neighboring lines (gratings) were found to be overlapped and above m ≧ 4 (line distance ≧ 800 nm), separated lines were fabricated.


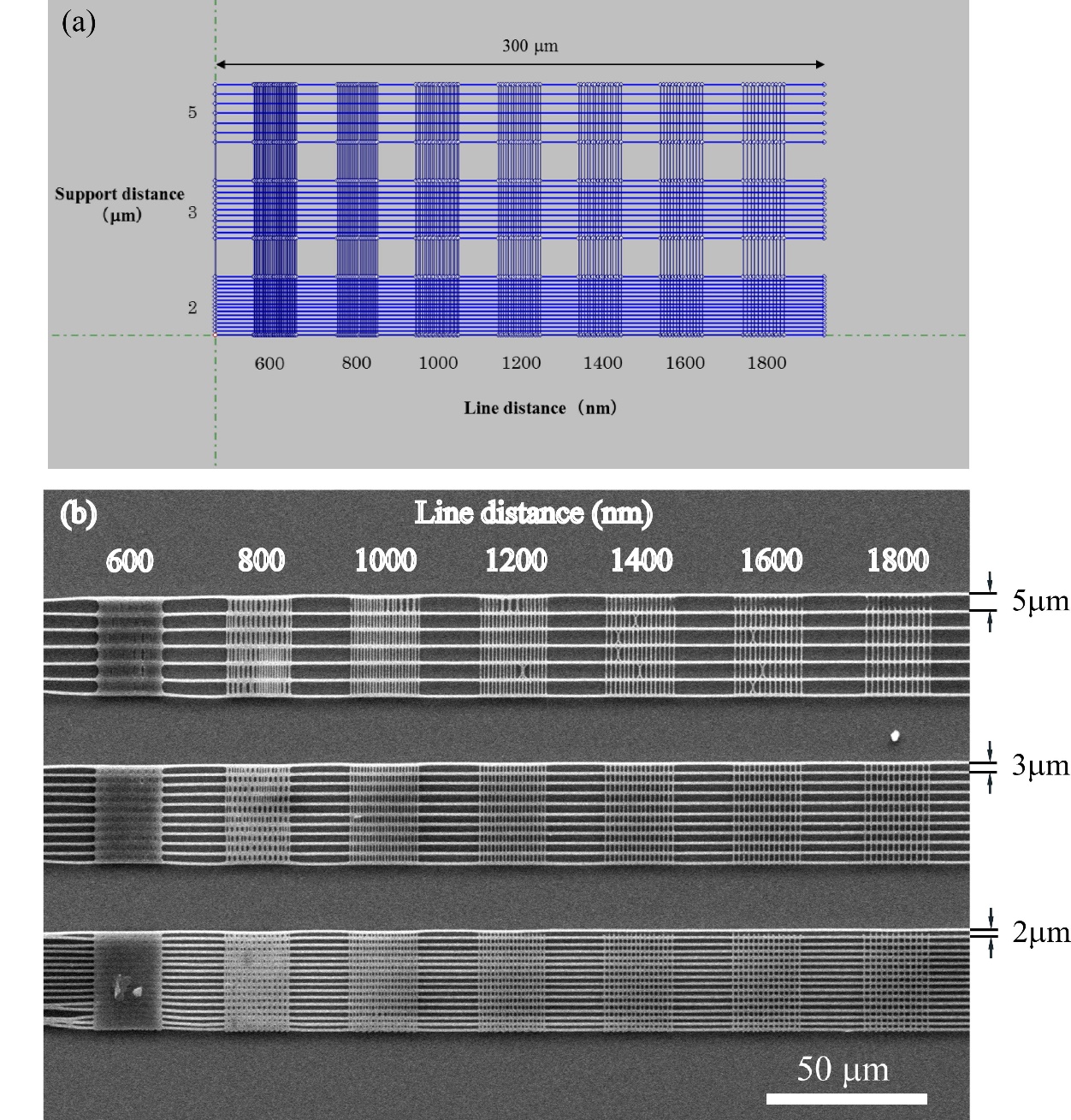


**Figure S2.** The effect of the interval of support walls on the grating structures fabricated.

**Waveguide Parameters**

The optical confinement of light in the waveguide and the proper effective index are important parameters for the waveguide laser. NL Guide software was used to calculate these parameters in four layer waveguide of air/R6G doped CA/SU-8/quartz substrate. Using refractive index and thickness of each layer summarized in **Table S2**, the optical confinement and effective refractive index are plotted as function of thickness of active layer in **Figure S3**.

**Table S2.** Refractive index at 583.6 nm and thickness of each layer (four layers).

| Material of layer | Refractive index at 583.6 nm | Thickness (µm) |
| --- | --- | --- |
| Air | 1.000 | 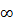 |
| R6G doped CA | 1.486 | ***x***  0.4, 0.6, 0.8, 0.9, 1.25, 1.7 |
| SU-8 | 1.604 |
| Quartz substrate | 1.457 | 1000 |


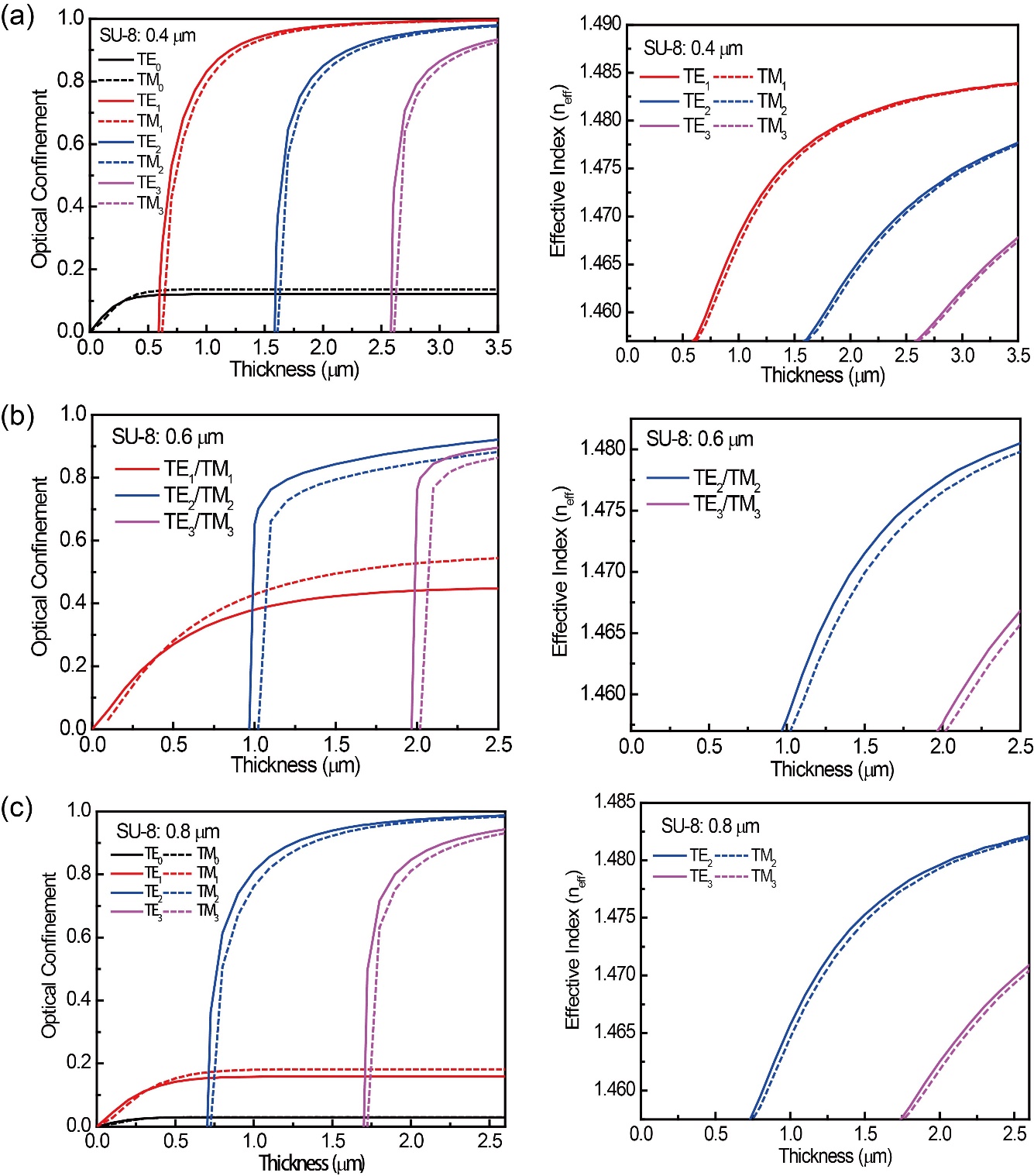


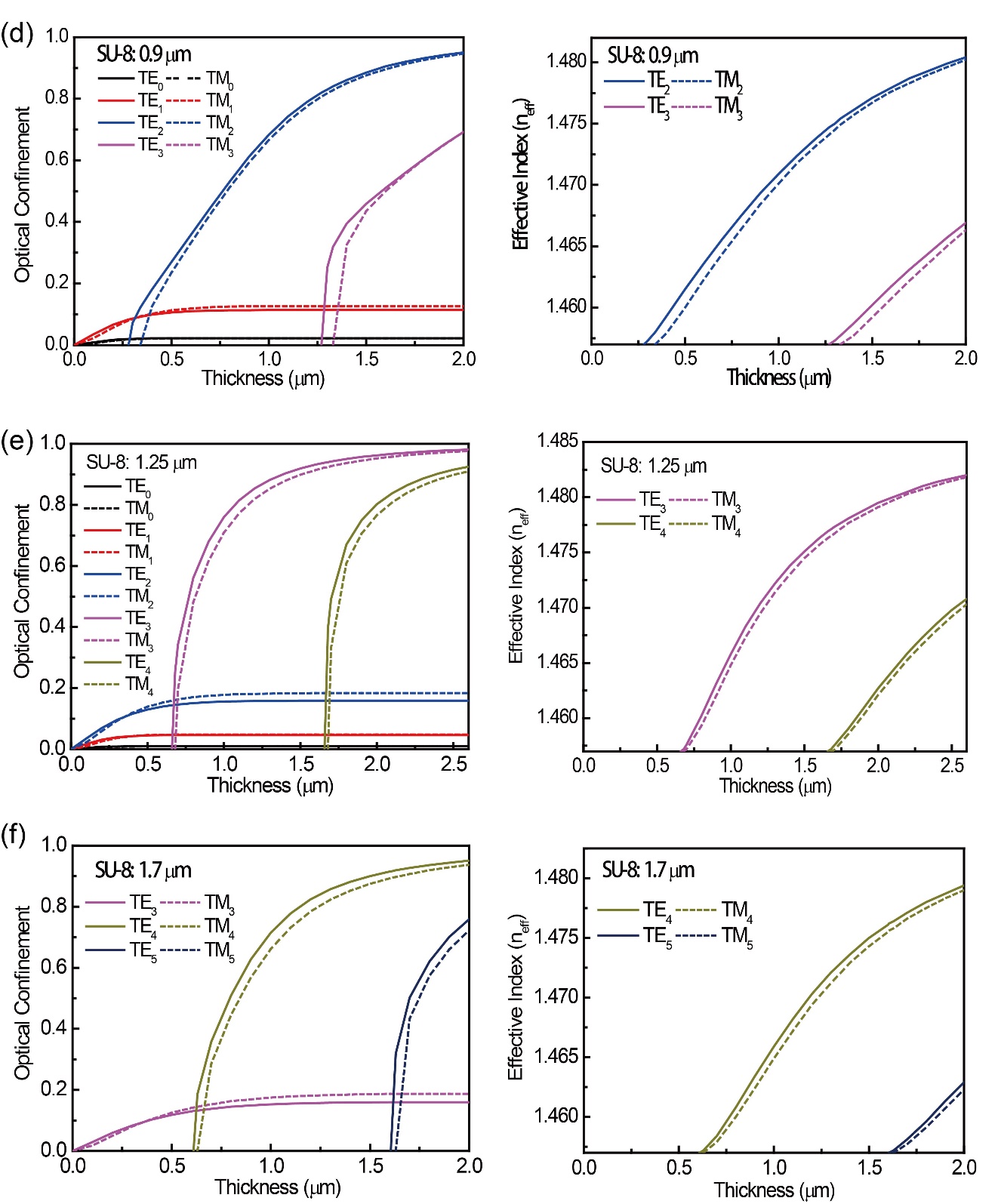


**Figure S3.** The plots of the optical confinement and the effective refractive index as function of thickness of laser active layer.
